# Breaking down the RECIST 1.1 double read variability in lung trials: What do baseline assessments tell us?

**DOI:** 10.3389/fonc.2023.988784

**Published:** 2023-03-16

**Authors:** Antoine Iannessi, Hubert Beaumont

**Affiliations:** Imaging Lab, Median Technologies, Valbonne, France

**Keywords:** clinical trial, variability, RECIST, computed tomography, lung cancer

## Abstract

**Background:**

In clinical trials with imaging, Blinded Independent Central Review (BICR) with double reads ensures data blinding and reduces bias in drug evaluations. As double reads can cause discrepancies, evaluations require close monitoring which substantially increases clinical trial costs. We sought to document the variability of double reads at baseline, and variabilities across individual readers and lung trials.

**Material and methods:**

We retrospectively analyzed data from five BICR clinical trials evaluating 1720 lung cancer patients treated with immunotherapy or targeted therapy. Fifteen radiologists were involved. The variability was analyzed using a set of 71 features derived from tumor selection, measurements, and disease location. We selected a subset of readers that evaluated ≥50 patients in ≥two trials, to compare individual reader’s selections. Finally, we evaluated inter-trial homogeneity using a subset of patients for whom both readers assessed the exact same disease locations. Significance level was 0.05. Multiple pair-wise comparisons of continuous variables and proportions were performed using one-way ANOVA and Marascuilo procedure, respectively.

**Results:**

Across trials, on average per patient, target lesion (TL) number ranged 1.9 to 3.0, sum of tumor diameter (SOD) 57.1 to 91.9 mm. MeanSOD=83.7 mm. In four trials, MeanSOD of double reads was significantly different. Less than 10% of patients had TLs selected in completely different organs and 43.5% had at least one selected in different organs. Discrepancies in disease locations happened mainly in lymph nodes (20.1%) and bones (12.2%). Discrepancies in measurable disease happened mainly in lung (19.6%). Between individual readers, the MeanSOD and disease selection were significantly different (p<0.001). In inter-trials comparisons, on average per patient, the number of selected TLs ranged 2.1 to 2.8, MeanSOD 61.0 to 92.4 mm. Trials were significantly different in MeanSOD (p<0.0001) and average number of selected TLs (p=0.007). The proportion of patients having one of the top diseases was significantly different only between two trials for lung. Significant differences were observed for all other disease locations (p<0.05).

**Conclusions:**

We found significant double read variabilities at baseline, evidence of reading patterns and a means to compare trials. Clinical trial reliability is influenced by the interplay of readers, patients and trial design.

## 1 Highlights

- In RECIST BICR trials with double reads there is large variability in tumor measurement and localization.
- Individual reader’s assessments are significantly different
- Advanced lung cancer trials with similar treatments can be significantly different in terms of baseline assessments.

## 2 Background

Since 2004 (1, 2), Blinded Independent Central Review (BICR) with double reads has been promoted in clinical trials with imaging to ensure data blinding and to reduce bias (3). A direct consequence of double reads is inter-reader variability. Because of these variabilities, discrepancies in the evaluation of treatment response during trials with double reads need to be monitored and, eventually, be adjudicated by a third reader (4). This directly impacts the quality and the cost of clinical trials that all stakeholders strive to mitigate. A better understanding of the root causes of the variability is needed. The ability to trigger warnings as early as after baseline evaluations would help reduce inter-reader variabilities during trial monitoring.

In clinical trials for drug development, the discrepancy rate of the treatment response assessment is the preferred indicator that summarizes the reliability of treatment evaluation (5). However, the discrepancy rate is a high-level indicator that encompasses all possible root causes of variability including the technical variability of image acquisition (6) and the interpretation of images (aka. reader variability). To manage variability, standardized reading rules are applied to radiology assessments that quantify the response: the response evaluation criteria, i.e. Response Evaluation Criteria in Solid Tumors (RECIST).

Several criteria-derived variability factors have been documented (7, 8). A large proportion of discrepant responses originate due to the subjectivity of the baseline assessment (9, 10) when using RECIST (11). Indeed, many imaging response criteria are based on the relative modifications from baseline, therefore it is logical that the initial definition of the disease has an impact on the response.

When endorsing an omnibus reference value of discrepancy rates based on the literature, the underlining assumption is that variabilities in reads, and the magnitudes of these variabilities, are consistent across “comparable” trials (12) however, this assumption has not been clearly confirmed. Indeed, less attention was given to the variability caused by the initial disease presentation and the heterogeneity of recruited readers across so called “comparable” trials.

In this paper, we consider similar lung clinical trials and focus on the baseline analysis of imaging data. We describe the distribution of double read variabilities, and compare the specificities of assessments between readers and, for individual readers across trials.

## 3 Methods

### 3.1 Study data

We aimed at minimizing the heterogeneity of our data by selecting studies with “comparable” indication and inclusion criteria. Our retrospective analysis included assessment data from five BICR clinical trials (Trials 1-5) that evaluated immunotherapy or targeted therapy for lung cancer. The selected BICR trials were conducted between 2017 and 2021 and used double reads with adjudication based on RECIST 1.1 guidelines. All data were fully blinded for sponsor data, study protocol number, therapeutic agent, subject demographics, and randomization. For these five trials, a total of 1720 patients were evaluated by 15 Board Certified US and Europe, 10y+ Senior Radiologist with previous experience in central RECIST 1.1 assessment (Reader R1-R15) (**Table 1**). The central reads were all performed using the same radiological reading platform (LMS; Median Technologies, France) ensuring automatic data extraction for analysis.

**Table 1:** Description of included trials.

Trial ID	Indication	Phase	Therapy	Study specific criteria (protocol and read rules)	Readers ID
**Trial 1**	Metastatic NSCLC	III	Immune checkpoints + chemotherapy *vs*. Chemotherapy + placebo	Measurable disease No central eligibility Brain metastases can only be non-targe lesions	R2, R4, R5
**Trial 2**	Metastatic NSCLC	III	Immune checkpoints + chemotherapy *vs*. Chemotherapy + placebo	Measurable disease Central eligibility process Brain metastases can only be non-target lesions	R4, R5, R6
**Trial 3**	Metastatic NSCLC	II	Tyrosine kinase inhibitors	Measurable disease No central eligibility Brain metastases can be target lesion	R1, R2, R6, R7
**Trial 4**	Metastatic NSCLC	III	Tyrosine kinase inhibitors	Measurable disease No central eligibility Brain metastases can be target lesion	R1, R3, R5, R7
**Trial 5**	Metastatic NSCLC	III	Immune checkpoints + chemotherapy *vs*. Chemotherapy + placebo	Measurable disease No central eligibility Brain metastases can be target lesion	R2, R3, R5, R7

Primary study endpoints were: Progression Free Survival (PFS) and Overall Response Rate (ORR). Patients were treated for Metastatic Non-Small Cell Lung Cancer (NSCLC).

### 3.2 Independent central review

The pool of 15 independent radiologists reading across the five selected trials were trained on the RECIST 1.1 criteria and study protocol inclusion criteria regarding brain metastasis to perform a BICR of each baseline image and to determine the radiologic timepoint response in accordance with these read rules. In each trial, the radiologist roles (i.e., independent reader IR1 or IR2) were randomly assigned to a Reader ID (R1 to R15) at the onset of the trial to create a double reading paradigm. All images and readers annotations underwent a quality control (e.g., checking the conformance with RECIST guideline and to the review protocol of the study) using software and operated by dedicated staff before the patient response to be evaluated. To improve the reliability of evaluations, the double reading paradigm involve a third reader when readers disagree on patient response, even at the early steps of eligibility.

Following RECIST 1.1 criteria, the tumor burden is quantified by the sum of diameter (SOD) as the sum of the largest lesions selected as targets lesions (TLs) within the “measurable” disease. To be measurable and qualify for a TL, the finding must measure at least 1cm for solid tumor or 1.5 cm for lymph-nodes. To be representative of the metastatic disease extent, the selection should be distributed across all involved organs and avoid the priorly irradiated areas. This prior therapy information was provided to the central readers. In total, a maximum of five TLs, maximum two per organ, are selected at baseline. Then, any additional lesions, smaller lesions and truly non-measurable lesions (e.g., blastic bone lesions) are represented by selecting Non-Target Lesions (NTL) which are only qualitatively assessed. For diffuse disease, the NTL lesions can be grouped instead of itemizing each one of the metastases.

### 3.3 RECIST 1.1 assessment analysis

#### 3.3.1 Initial bivariate analysis

**Figure 1:**
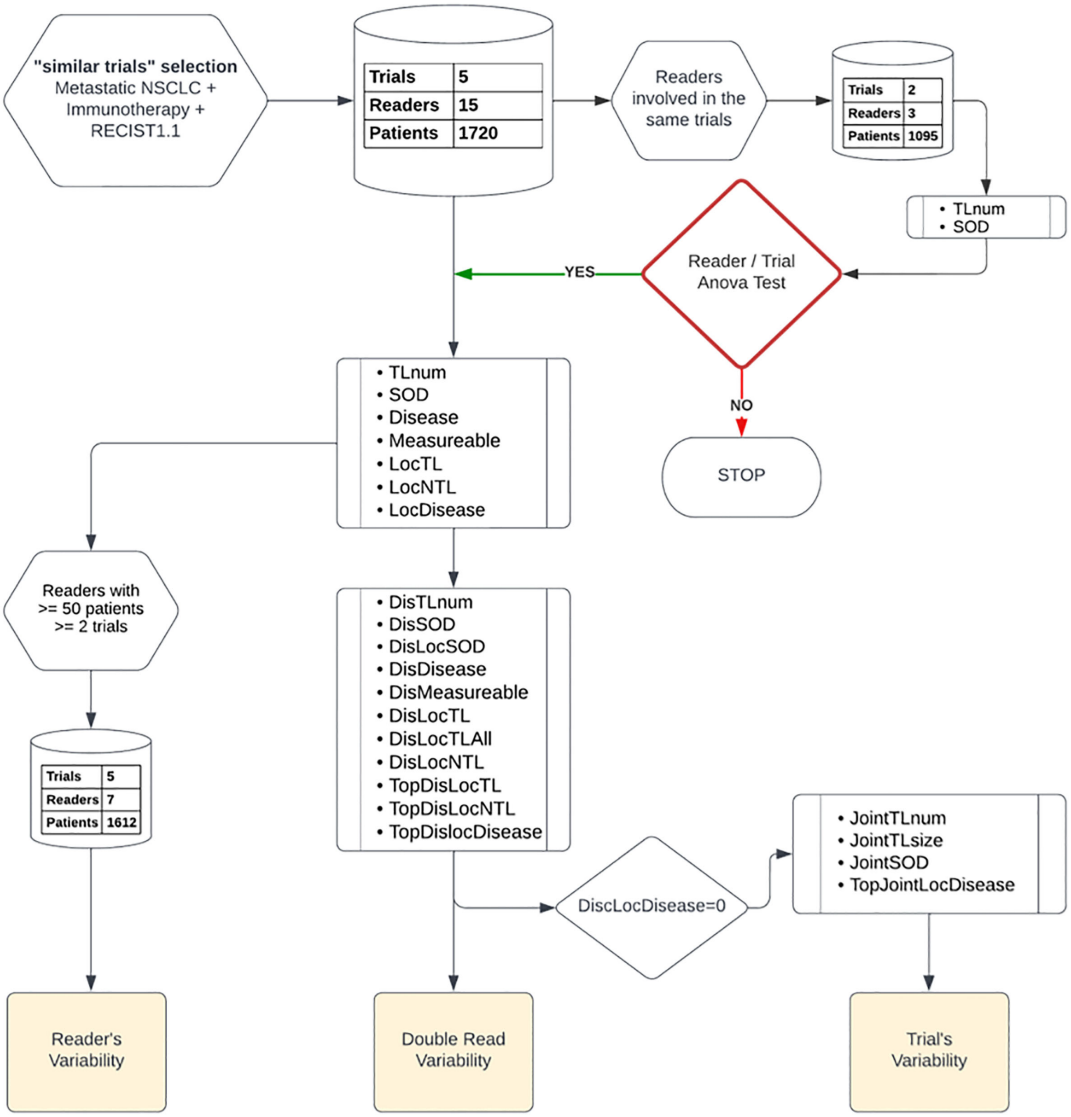
Data analysis plan: Top down, we started by a two-way factor analysis of Reader and Trial, then we documented readers, double-read, and trials’ variabilities. We report, from top to bottom, the type of features and data preparation involved in each step of our analysis.

The study plan is depicted in **Figure 1**. Initially, to ensure the validity of our subsequent results, we first considered a subset of readers who participated in the same subset of trials, then we checked that the variability between the readers was not linked to the studies in which they were involved. Also, that the variability between studies was not related to the readers who carried them out. We effectively measured the bi-factorial impact of reader~trial interaction on the variability of SOD and the number of selected TLs at baseline through a two-way analysis of variance.

#### 3.3.2 Core analysis: variability according to three perspectives

The RECIST 1.1 baseline assessment provides quantitative and qualitative information on disease extent and its spread throughout organs. Accordingly, we based our variability analysis on features designed to report on the extent and spread of the disease.

As detailed below, our analysis was divided into three parts:

##### 3.3.2.1 Double read variability

**Figure 2:**
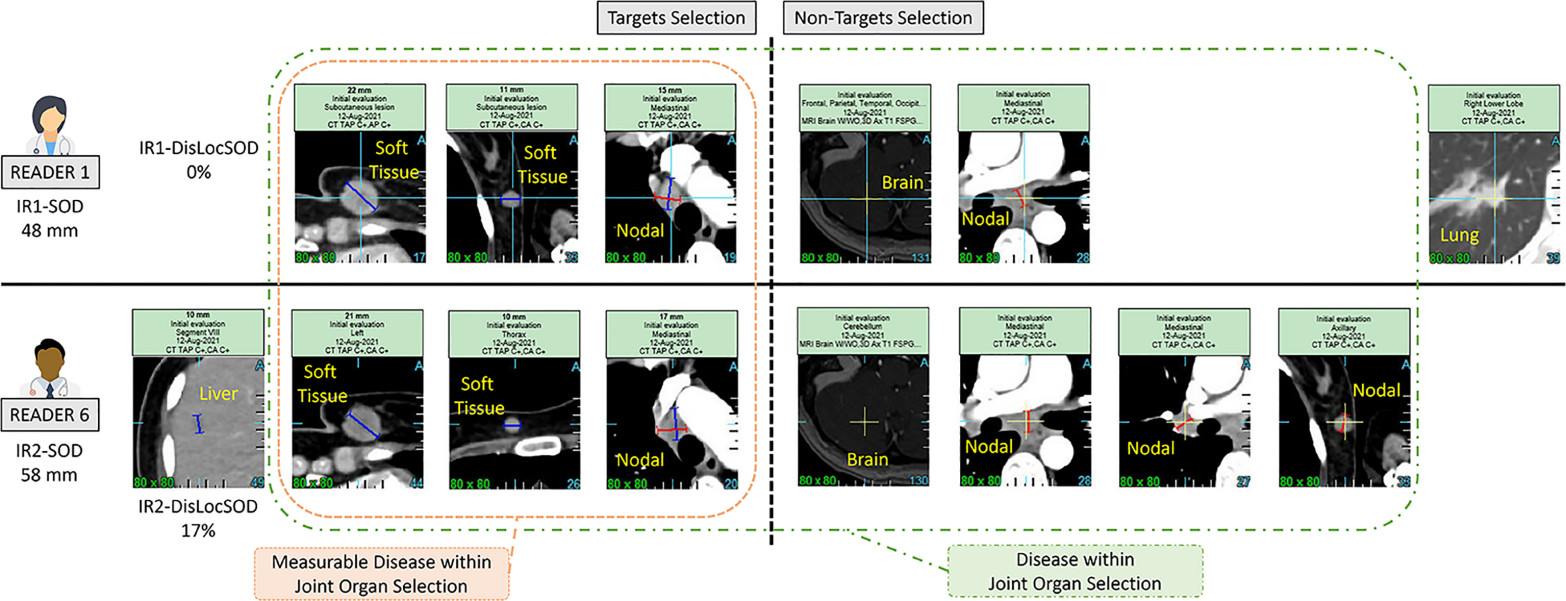
Example of a double read baseline disease assessment by RECIST 1.1. One patient in Trial 3. Each reader’s selection is illustrated and grouped inside a Venn diagram illustrating the common organ selection of the disease and its measurable part. The discrepant selection within the measurable SOD illustrates the meaning and calculation of the double read DisLocSOD feature as (IR1-DisLocSOD + IR2-DisLocSOD)/2. The reader 6 identified disease in the liver and measured a centimetric liver nodule that could have been considered equivocal by reader 1.

We investigated inter-reader variability in the original double read setting through a set of predefined disease-related quantitative and location features (double read-derived features shown in **Table 2**, disease locations listed in Annex A). These features describe the two readers’ selection of patients’ tumors (TLs and NTLs) at baseline as illustrated in **Figure 2**. We documented the distribution of these features and compared them across trials. We provided typical values.

**Table 2:** Double read-extracted features.

Discrepancy analysis (averaged per double reads)	
***DisTLnum***	Difference in the number of TLs recorded during double reads
***DisSOD***	Difference of SOD recorded during double reads (proportional difference of double read SOD in %)
***DisLocSOD***	SOD of TL belonging to discrepant selected organs in double reads (ratio is derived by dividing by the reader total SOD value in %). Computed when one of the readers reported measurable disease
***DisDisease***	Proportion of patients reported with no disease at all by one of the readers (no TL and no NTL at all for one reader)
***DisMeas***	Proportion of patients reported with disease by both readers but one of the readers reported no measurable disease (No TL but at least one NTL)
***DisLocTL***	Proportion of patients for which readers targeted at least one TL at a different disease location
***DisLocTLAll***	Proportion of patients for which readers targeted all their TLs in totally different locations
***DisLocNTL***	Proportion of patients for which readers targeted at least one NTL at a different disease location
***TopDisLocTL***	List of the most represented discrepant TL locations
***TopDisLocNTL***	List of the most represented discrepant NTL locations
***TopDisLocDisease***	List of the most represented discrepant disease locations
Measurements derived from jointly selected organs (averaged per trial) when DisLocDisease = 0	
***JointTLnum***	Number of TL recorded in non-discrepant diseased organs
***JointTLSize***	Size of the TL recorded in non-discrepant diseased organs
***JointSOD***	Tumor burden recorded in non-discrepant diseased organs
***TopJointLocDisease***	List of the most represented non-discrepant diseased location (TL or NTL)

Feature acronyms and definitions that were used for the description of disease selection variability at baseline.

##### 3.3.2.2 Reader’s variability

To further compare reader’s selections, we selected a subset of readers that evaluated 50 patients or more in two or more trials (i.e. more than 100 evaluations by an individual reader), therefore lowering the weight of the “trial” and “patient” covariates. For each reader, we computed the average number of TLs and SOD per patient (single reader-derived features shown in **Table 3**) and the proportion of patients in the most represented discrepant disease location (TopDisLocDisease, see **Table 2**) as previously determined in our double read variability analysis. We compared these baseline selection features with each other.

Table 3Single read-extracted features.

**Table:** 

Quantitative features	
***TLNum***	Number of TLs recorded per patient
***TLSize***	SOD/TLNum per patient (in mm)
***SOD***	Tumor burden as the Sum of tumor Diameter per patient (in mm)

**Table:** 

Qualitative features	
***Disease***	Proportion of patients recorded with disease (at least one TL or NTL)
***Meas***	Proportion of patients recorded with measurable disease
***LocTL***	Proportion of patients recorded with TL located in a specific organ
***LocNTL***	Proportion of patients recorded with NTL located in a specific organ
***LocDisease***	Proportion of patients recorded with TL *or* NTL located in a specific organ

SOD, Sum of Diamters; TLNum, Number of target lesion; TL, Target lesion; NTL, Non target lesion.

Feature acronyms and definitions that were used for the description of disease selection at baseline per reader.

##### 3.3.2.3 Trial’s variability

The five trials selected were deemed “comparable” as they evaluated advanced lung cancer treated with similar therapeutics. The third part of our study checked the validity of our assumption by analyzing inter-trial homogeneity using the subset of patients for whom both readers selected the same disease locations (either TLs or NTLs). We assumed that, as both readers agreed on disease location (as in **Figure 2**), the derived findings would be more reliable, therefore allowing a more relevant inter-trial comparison. For this purpose, for each of the three quantitative features related to tumor burden (**Table 2**), we averaged the joint double read measurements. Similarly, we computed the proportion of patients for whom the top five metastatic locations were reported in agreement during double reads.

### 3.4 Statistics

All statistics were performed using base version and packages from R CRAN freeware. Level of significance was set to 5%. Continuous variables were analyzed using a paired two sample non-parametric Wilcoxon test. The confidence interval of the mean difference was computed using the “misty” package. A violin plot was used to display the difference of measurements. Multiple pair-wise comparisons between the five trials and between the subset of seven readers were performed. For comparison of continuous variables, when assumptions for homoscedasticity (Levene’s test, “base” package) and normality (Jarque–Bera test, “lawstat” package) were met, one-way analysis of variance (ANOVA) (“base” package) was used. When these assumptions were not met, multiple comparison was performed using the Kruskal-Wallis test. Multiple comparison for proportions was performed according to Marascuilo procedure (13).

Type III two-way ANOVA (“car” package) was performed after BoxCox transformation of the measure and homoscedasticity and normality of residuals checked using Bartlett’s and Shapiro-Wilk’s test, respectively.

## 4 Results

As a preliminary analysis, we considered three readers that were all involved in the same two trials with a total of 1095 patients (see **Figure 1**). We confirmed that reader and trial are both factors contributing to the variability of SOD and the number of selected TLs (p<0.0001). The interaction between the two main factors was not significant for SOD (p=0.24) or the number of selected TLs (p=0.67), meaning that, for our data, inter-reader variability had no effect on the measurement of inter-trial variability and vice versa.

### 4.1 Double read variability

The differences between reader’s measurements are summarized in the **Table 4**.

**Table 4:** Double-read measurements (test of differences).

	DisDisease	DisMeas	Read-TLnum	Read-SOD (mm)	Read-DisLocSOD (%)
**Trial 1 *(N=333)***	0%	4.8% (16/333)	IR1-TLNum=2.1 ** IR2-TLNum=2.3	IR1-SOD=77.7 ** IR2-SOD=86.7	IR1-SPropSOD=19.2 ** IR2-SPropSOD=12.6
**Trial 2 *(N=493)***	0%	0.4% (2/493)	IR1-TLNum=1.9 ** IR2-TLNum=2.3	IR1-SOD=89.4 IR2-SOD=91.9	IR1-SPropSOD=10.7 ** IR2-SPropSOD=15.6
**Trial 3 *(N=240)***	0.8% (2/240)	23.1% (55/238)	IR1-TLNum=2.1 * IR2-TLNum=2.4	IR1-SOD=57.1 ** IR2-SOD=75.4	IR1-SPropSOD=14.9 * IR2-SPropSOD=18.7
**Trial 4 *(N=276)***	0%	6.1% (17/276)	IR1-TLNum=2.4 ** IR2-TLNum=3.0	IR1-SOD=71.3 ** IR2-SOD=78.4	IR1-SPropSOD=15.3 ** IR2-SPropSOD=21.4
**Trial 5 *(N=378)***	0%	1.3% (5/378)	IR1-TLNum=2.4 ** IR2-TLNum=2.6	IR1-SOD=91.0 IR2-SOD=89.9	IR1-SPropSOD=10.4 IR2-SPropSOD=10.9
**Average** **(N=1720)**	0.1% [0.01; 0.4]	5.5% [4.5; 6.7]	2.34 [2.29; 2.40]	83.7 [81.6; 85.8]	14.4% [13.1; 15.6]

We documented double read features (displayed by column) for the five clinical trials (displayed by row). The two left-most columns display discrepancy features. Only for patients reported as having measurable diseases, the three right-most columns display the means of each reader’s measurements (independent reader [IR]1 and 2), the p-value of the corresponding two-sample test of difference is indicated by asterisks: **, p<0.001; *, p<0.05; no asterisk means no statistically significant difference. The last row is the average overall measurements of both R1 and R2 with corresponding confidence intervals.

Regarding eligibility, our analysis showed a very low discrepancy rate for disease detection at baseline with an overall discrepancy rate <0.1%. The measurement of tumor burden was more variable with an overall discrepancy rate of approximately 6% for studies without a centralized eligibility process.

**Figure 3:**
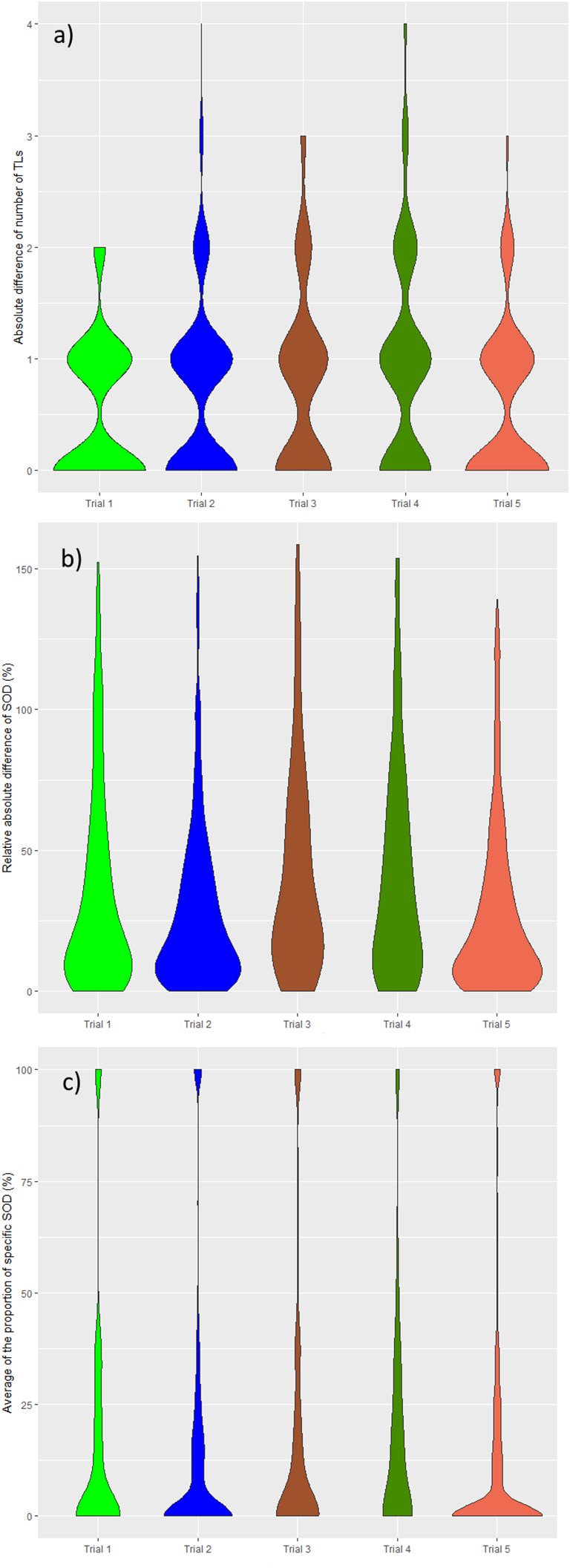
Distribution of double read measurement discrepancies. Distributions of double read measurement discrepancies are presented by color, representing each trial. Left to right; **(A)** the difference in the number of selected TLs (DisTLnum), **(B)** the proportional difference in SOD measurement (DisSOD), **(C)** the average of the inter-reader mean of readers (DisLocSOD).

In all trials the two pools of readers (IR1 and IR2) selected a statistically significantly different average number of TLs per patient, ranging from 1.9 to 3.0 across trials (median values being either 2 or 3). The average SOD per patient ranged from 57.1 mm to 91.9 mm across trials with an overall average SOD of approximately 84 mm. The difference in the number of TLs during double reads (DisTLnum) was often higher than 2 and could be as high as 4 (**Figure 3A**). The difference in SOD (The absolute difference of SOD divided by the average of the double reads SOD in %, DisSOD) reached more than 100% in all trials (**Figure 3B**).

In all trials, except Trial 5, average tumor burden was statistically significantly different between the two readers.

The average specific proportional SOD (DisLocSOD) ranged from 10.4% to 21.4%. In all trials, except Trial 5, DisLocSOD was statistically significantly different between the two readers.

The distribution of double read discrepancies in TL measurements are depicted in **Figure 3** as violin plots, which confirm the findings in **Table 4**. **Figure 3A** shows that the DisTLnum was different across trials (median value for DisTLnum was 1 for Trial 1 and 5 and was 2 for the other trials). Per patient, the same number of TLs were selected in 49.6%, 46.1%, 27.3%, 33.4% and 53.0% of the Trial 1 to 5, respectively. These proportions were significantly different across trials (Marascuilo procedure, p<0.05). **Figure 3B** shows that for all trials, the DisSOD was higher than 100%, reaching 150% in all except Trial 5. The mean absolute DisSOD was 34.6%, 27.4, 41.2%, 40.6% and 26.8% in Trial 1 to 5, respectively. Therefore, three trials had an absolute difference significantly higher than 33%. **Figure 3C** shows that for all trials the average DisLocSOD value can reach 100%, which is confirmed by the proportion of patients for which readers targeted all TLs in totally different locations (DisLocTLAll) being different from zero for all trials (see **Table 5**). For Trials 1 to 5, 75% of their tumor burden had an average DisLocSOD value less than 22.2%, 15.8%, 19.9%, 26.3% and 12.2%, respectively. Therefore, the average DisLocSOD value of the third quartile in a trial can be two times higher than in another trial.

**Table 5:** Distribution of discrepancies in disease locations.

*Trial ID*	Measurable disease (%)			Non-measurable disease (%)		Disease (%)
	DisLocTL	DisLocTLAll	TopDisLocTL	DisNTLLoc	TopDisLocNTL	TopDisLocDisease
**Trial 1** **(N=333)**	42.9 (136/317)	7.5 (24/317)	Lung: 25.2 LN: 19.2 Pleura: 3.9 Bone: 3.3 Liver: 0.9	61.6 (205/333)	LN: 34.8 Lung: 31.8 Bone: 7.2 Pleura: 4.8 Liver: 3.6	LN: 21.9 Lung: 10.8 Bone: 8.1 Pleura: 6.3 Adrenal:2.7 Liver: 1.8
**Trial 2 (N=493)**	40.9 (201/491)	6.3 (31/491)	LN: 24.1 Lung: 13.2 Pleura: 3.1 Adrenal: 3.1	66.9 (330/493)	Lung: 32.0 LN: 30.0 Bone: 13.2 Pleura: 12.2 Liver: 3.0	LN: 25.8 Pleura: 12.6 Bone: 12.6 Lung: 8.1 Adrenal: 4.0
**Trial 3** **(N=240)**	46.9 (86/183)	8.2 (15/183)	Lung: 22.1 LN: 19.6 Brain: 11.2 Pleura: 6.7	64.2 (154/240)	Lung: 30.4 LN: 21.7 Pleura: 15.4 Bone: 11.25 Liver: 6.6	LN: 10.6 Pleura: 15.8 Lung: 14.2 Bone: 11.2 Liver: 6.7
**Trial 4** **(N=276)**	57.9 (150/259)	5.8 (15/259)	Lung: 27.2 LN: 21.7 Misc: 16.7 Brain: 9.8 Liver: 6.5	73.5 203/276	LN: 33.7. Lung: 28.9 Misc: 19.2 Bone: 18.1 Brain: 12.3	LN: 23.2 Misc: 21.0 Bone: 17.4 Lung: 13.4 Brain: 10.4
**Trial 5** **(N=378)**	36 (133/373)	4 (15/373)	Lung: 14.0 LN: 12.4 Pleura: 6.6 Bone: 4.8	60.5% (229/378)	Lung: 38.6 LN: 20.6 Bone: 10.8 Pleura: 4.8	LN: 14.8 Bone: 12.4 Lung: 6.9 Pleura: 6.1
**Overall** **(N= 1720)**	43.5 [41.0; 45.9] (706/1623)	6.1 [5.0; 7.4] (100/1623)	LN: 19.6 Lung:19.2 Pleura: 4.0 Misc: 3.3	65.2 [62.9; 67.4] (1121/1720)	Lung: 32.7 LN: 28.3 Bone: 12.0 Pleura: 7.6	LN: 20.1 Bone: 12.2 Lung: 10.1 Pleura: 8.4

LN, Lymph node; TL, Target lesion; NTL, Non target lesion.

For the five clinical trials (displayed in rows) we computed: The proportion of patients for which readers targeted all their TLs in totally different locations (DisLocTLAll); the proportion of patients for which readers targeted at least one TL (DisTLLoc) or NTL (DisLocNTL) at a different disease location; the top proportion of discrepancies in TL (TopDisLocTL), NTL (TopDisLocNTL) and disease locations (TopDisLocDisease) (in % of patients concerned).

**Figure 4:**
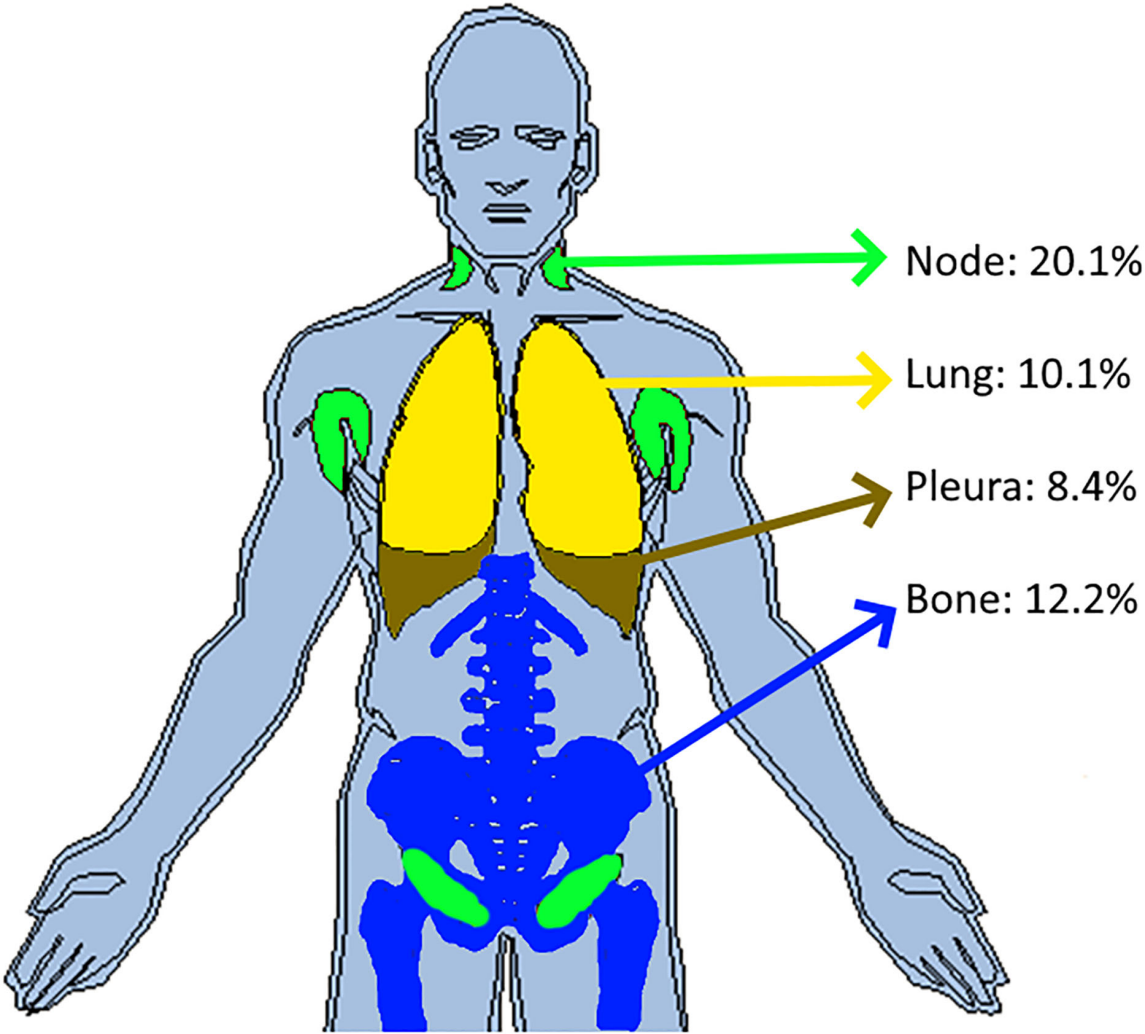
Inter-reader discrepancies in location of the disease. In pooling data from five comparable trials, we colored the top four organs where inter-reader discrepancy occurred in associating the corresponding proportion of patient concerned. Top four organs were the lymph nodes (20.1%), bones (12.2%), lungs (10.1%) and pleura (8.4%).

The distribution of reader’s discrepancies according to disease location is summarized in **Table 5**. For all trials, a non-null proportion of patients had TLs selected in completely different organs, however, this proportion concerned less than 10% of patients. Overall, the readers targeted at least one different organ (TLs) in 43.5% of patients, ranging from 36.0% to 57.9% across trials. The organs with the highest risk of discrepancies were the lymph nodes (20.1%) and bones (12.2%) (see **Figure 4**). The discrepancies in detection of measurable disease occurred mainly in the lungs (19.6%).

### 4.2 Reader’s variability

Readers’ selections and measurement data from the five trials were pooled and are summarized in **Table 6**.

**Table 6:** Distribution of readers’ selections across trials.

Readers ID	Nb Pat	Quantification		Proportion of patients in top discrepant disease locations				
		TLNum	SOD (mm)	Nodal (%)	Bone (%)	Lung (%)	Pleura (%)	Infrequent (%)
**Reader 1**	253	1.6 [1.5; 1.8]	47.5 [42.2; 52.7]	52.6 [46.2; 58.9]	33.6 [27.8; 39.8]	79.8 [74.3; 84.6]	12.3 [8.5; 16.9]	20.6 [15.7; 26.0]
**Reader 2**	304	1.9 [1.8; 2.0]	60.3 [55.7; 64.9]	70.4 [64.9; 75.5]	5.9 [3.5; 9.2]	93.1 [89.6; 95.7]	3.6 [1.8; 6.4]	9.5 [6.5; 13.4]
**Reader 3**	414	2.5 [2.4; 2.7]	80.9 [76.5; 85.3]	82.1 [78.1; 85.6]	34.3 [29.7; 39.1]	88.9 [85.4; 91.7]	0 [NA; NA]	28.5 [24.2; 33.1]
**Reader 4**	106	2.2 [2.0; 2.4]	74.4 [67.4; 81.3]	88.7 [81.0; 94.0]	5.7 [2.1; 11.9]	71.7 [62.1; 80.0]	5.7 [2.1; 11.9]	0.0 [NA; NA]
**Reader 5**	734	2.7 [2.6; 2.8]	94.0 [90.6; 97.3]	74.9 [71.6; 78.0]	18.0 [15.3; 20.9]	96.2 [94.5; 97.4]	3.7 [2.4; 5.3]	8.8 [6.9; 11.1]
**Reader 6**	365	1.7 [1.6; 1.8]	79.6 [74.7; 84.4]	46.6 [41.3; 51.8]	20.8 [16.8; 25.3]	94.5 [91.7; 96.7]	15.3 [11.8; 19.4]	12.9 [9.6; 16.7]
**Reader 7**	423	2.4 [2.3; 2.6]	77.8 [82.4; 73.1]	65.2 [60.5; 69.8]	25.1 [21.0; 29.5]	87.0 [83.4; 90.0]	0.2 [0.0; 1.3]	21.7 [17.9; 26.0]

SOD, Sum of Diamters; TLNum, Number of target lesion.

For seven readers (displayed in rows) involved in two or more trials, we reported in column 1) the number of assessed patients at baseline, 2) the average number of TLs selected in patients, 3) the average measured SOD, 4) the proportion of patients for whom nodal, bone, lung, pleura and infrequent disease were evaluated. Confidence intervals are provided in brackets.

**Figure 5:**
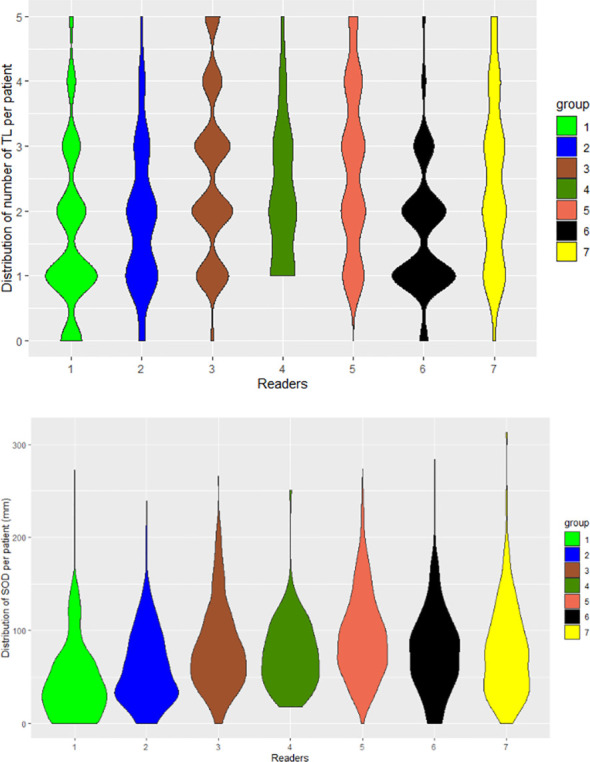
Distribution of readers’ measurements. The seven readers are represented by violin plots for the number of selected TLs (TLNum) per patient (left) and SOD measurements (right).

Derived from the data in **Table 6**, and graphically confirmed in **Figure 5**, the distribution of readers’ SODs and TLNum were statistically significantly different (Kruskal-Wallis, p<0.001). To be noted: for one patient in Trial 3, two readers (R1 and R6) did not find any measurable disease.

**Figure 6:**
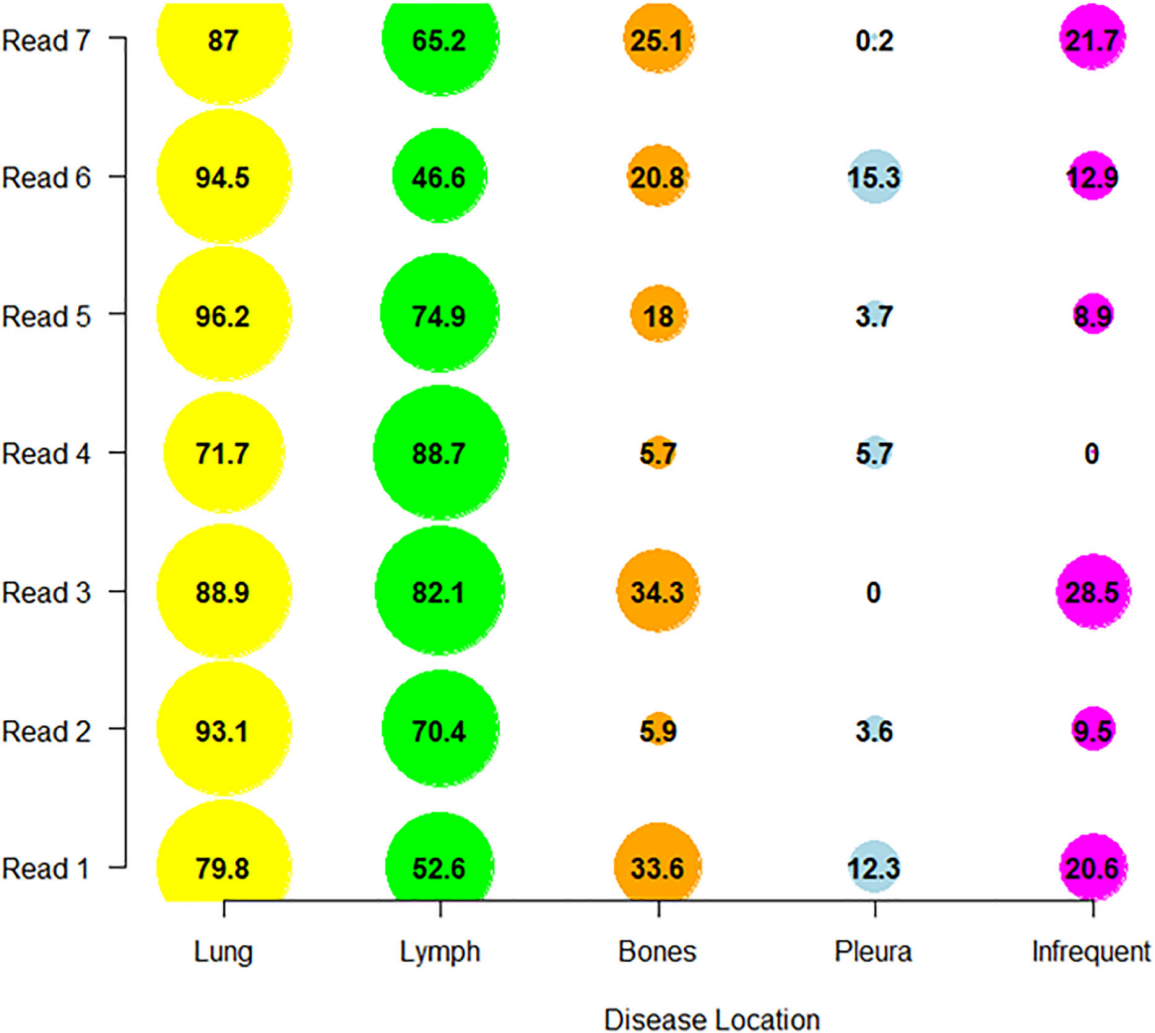
Distribution of reader’s selections. We display per reader, the proportion of patients (as percentage) in which the readers collected the disease in one of the top four discrepant locations (derived from **Table 6**) and in infrequent locations.

A 21 pair-wise comparison of the seven readers showed that 14, 13, 13, 10 and 10 pairs of readers (out of 21) significantly differed in the proportion of patients for whom diseases were selected in nodal, bones, infrequent (14) (see Annex A for definition), lung and pleura disease locations, respectively (Marascuilo procedure). These differences in proportion are depicted in **Figure 6**.

### 4.3 Trial’s variability

**Figure 7:**
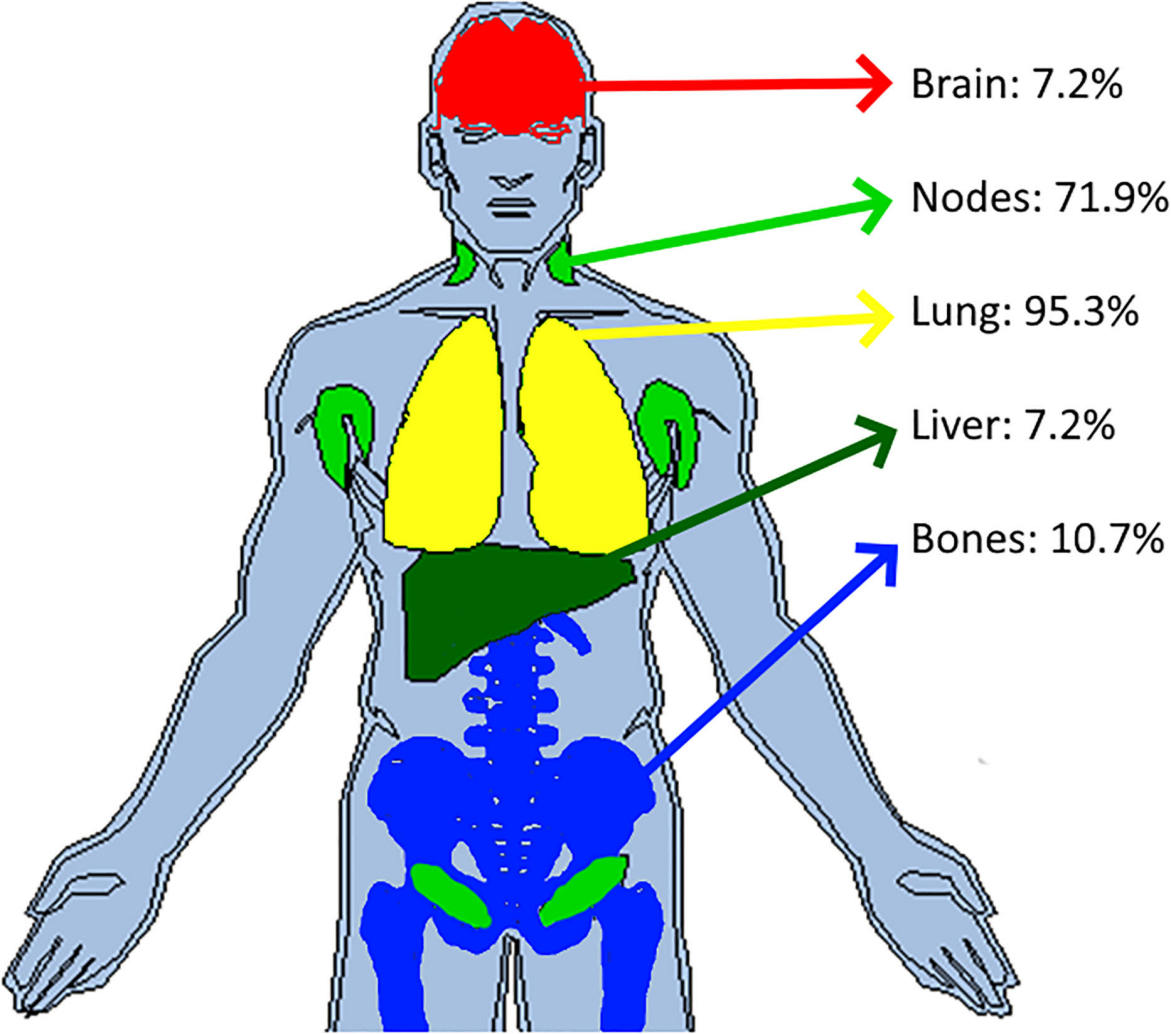
Top jointly selected organs. In pooling data from five comparable trials, we colored the top four jointly selected organs. With attached proportion of patient concerned, top jointly selected organs were the lungs (95.3%), lymph nodes (71.9%), bones (10.7%), brain (7.2%) and liver (7.2%).

The following results are for the subset of patients for whom the two readers documented the same disease locations (**Table 7**). On average per patient, the number of selected TLs ranged from 2.1 to 2.8, SOD ranged from 61.0 mm to 92.4 mm and average TL size ranged from 28.0 mm to 44.9 mm. Multiple comparisons showed that trials differed in average SOD, the number of selected TLs and the average size of selected TLs (Kruskal-Wallis, p<0.0001). The proportion of patients having one of the top disease locations was statistically significantly different for lung between Trial 2 and 3 only. Multiple statistically significant differences were measured for all other disease locations (Marascuilo procedure, p<0.05). **Figure 7** displays the top jointly selected diseased organs.

**Table 7:** Trial features for double read with joint organ selection.

Trial ID	JointTLNum	JointTLSize (mm)	JointSOD (mm)	TopJointLocDisease
**Trial 1** ***(N=184/333)***	2.2 [2.1; 2.4]	40.3 [37.4; 43.3]	82.8 [76.9; 88.8]	Lung=94% LN=73.9% Liver=7.1% Brain=2.2% Bone=1.1%
**Trial 2** ***(N=224/493)***	2.2 [2.1; 2.4]	44.9 [42.4; 47.5]	92.4 [86.9; 97.9]	Lung=99.1% LN=69.2% Liver=5.8% Brain=2.2% Bone=6.7%
**Trial 3** ***(N=102/240)***	2.1 [1.9; 2.4]	29.4 [26.6; 32.2]	61.0 [52.8; 69.1]	Lung=87.2% LN=45.1% Liver=2.9% Brain=22.5% Bone=21.6%
**Trial 4** ***(N=100/276)***	2.8 [2.6; 3.1]	28 [25.8; 30.3]	78.4 [69.6; 87.1]	Lung=93% LN=75% Bone=36% Brain=22% Liver=14%
***Trial 5*** ***(N=223/378)***	2.6 [2.4; 2.7]	38.4 [36.2; 40.6]	91.3 [86.4; 96.2]	Lung=97.3% LN=83.8% Liver=7.6% Brain=2.7% Bone=6.3%

LN, Lymph node.

For each of the five trials (number of patients shown), we computed, per patient, the average number of TLs selected by the two readers (JointTLNum), the average TLs’ diameter (JointTLSize) in mm, the average sum of TLs’ diameter (JointSOD) in mm and the proportion of patients where the top five diseased locations were assessed. Averaged values are displayed with corresponding 95% confidence intervals.

## 5 Discussion

We found that reader and trial were significant factors of variability (without interaction) for SOD and number of TLs estimated at baseline. This outcome allowed the development of the following discussion.

### 5.1 Variability between readers in disease detection and its measurability

The first variabilities in reading images lies in the identification of the disease. In clinical trials, it is key to include patients with relevant disease and a quantifiable tumor burden as required for proper treatment response assessment (11). For this reason, except for adjuvant evaluation setting, trials evaluating a treatment response endpoint usually require RECIST 1.1 **“**measurable**”** disease at baseline for eligibility, meaning that the radiologist should identify at least one TL in the recorded lesions at baseline. The eligibility evaluation is usually performed on site before submitting baseline examinations of screened patients for central review. To mitigate the risks of disagreement between the screening site and central review, another option is to perform eligibility evaluation centrally by involving three readers. The two concordant readers are then kept for the RECIST 1.1 assessment. Central eligibility review was performed for Trial 2, which explains the low rate of discrepancies at baseline in double reads. It is interesting to note that if Trial 2 is excluded, there is still a low average discrepancy rate (<1% regarding non-detection of disease and **<**10% regarding measurability status of the disease at baseline). However, the magnitude of discrepancies fluctuates widely across the trials with measurable *vs*. non-measurable disease discrepancies reaching up to 23% in Trial 3. This may be partly explained by Trial 3 having patients with the smallest tumor burden average reported by the readers.

### 5.2 Variability between readers in disease burden measurements

To be representative of metastatic disease, baseline selection evaluations may involve quantifying tumor burden by number of TLs and related SOD.

For, respectively, the number of TLs and SOD, three and four out of the five trials, had significant double reads differences (p<0.001) (**Table 4**).

Approximately 50% of patients had a different number of TLs recorded during double reads. This value is similar to the 59% reported by Kuhl et al. (15). Regarding the distribution of this quantitative discrepancy, violin plots showed readers had most often recorded one (rarely two) TL more or less than the paired reader. The discrepancy in SOD was in average higher than 25%, with violin plots showing a large range of variation, up to 150% difference for all trials. There are several reasons for readers’ measurement discrepancies and our results shows a larger variability on lung organ in respect to lymph-node. Indeed, lung metastasis delineation can be subjective if the reader needs to separate adjacent atelectasis.

The magnitude of differences in SOD raises questions regarding the impact this has on follow-up and RECIST 1.1 response thresholds (11). We know from Sharma et al. (16) that the variability in SOD at baseline is a risk for discrepant responses but, while threshold values for detecting significant longitudinal SOD changes (in follow up) are proposed (17), none are proposed for critical values for differences in SOD with double reads at baseline. Darkeh et al. (18) showed the impact of the number of TLs selected on discordances. If we assume a direct correlation between variability in the number of selected TLs and SOD, the conclusions of Darkeh et al. and Sharma et al. are consistent. Baseline SOD is also reported as an independent prognostic biomarker, however, the magnitude of variability questions the reliability its use (19).

In documenting the extent of the SOD variability, we confirmed previous works, notably the permissiveness of RECIST (20) in the selection of lesions to include as TLs. For selecting a TL, its size is not the only criteria, conspicuity, vicinity and the number of other candidate TLs are some other numerous factors that are left to readers appreciation. This subjective choice can easily explain a 150% discrepancy in the SOD between two observers.

### 5.3 Variability in assessments of disease distribution

As metastatic patients have multi-organ disease, RECIST 1.1 recommends a representative selection of TLs across all involved organs to capture the extent of the disease. In this analysis, we introduced a new quantitative feature (DisLocDisease) to represent the proportion of disease burden measured in organs by only one of the two paired readers. At baseline, due to the central review setting, historical data are censored; therefore readers may subjectively select, more or less equivocal lesions, such as the small liver nodule in **Figure 2** (10).

On average, the DisLocSOD represented up to 20% of the SOD per trial per reader. The violin plots of DisLocSOD displayed bimodal distributions where the second local maxima in the probability density function at 100% corresponds to the 6% of the patients assessed with zero common disease locations during double reading (DisLocTL). This discrepancy in disease location/measurement mainly involved assessments of lung and lymph node disease. The latter can be explained due to the “size” related threshold (short axis >=1.5 cm) of a measurable adenopathy according to RECIST 1.1. Specifically for our indication of interest, in the mediastinum of smoker patients, it is not uncommon to observe centimetric nodes which are otherwise non-specific (21) and not captured as TLs by the readers.

For double read assessments of NTLs, the bones were identified as the third most at risk location for discrepant metastatic disease identification. Indeed, bone metastases are almost always recorded as NTL as blastic lesions are truly non-measurable and even when they are measurable, RECIST 1.1 rules consider them as a secondary choice. We suspect that identification of bone metastasis demonstrates a variability during double reads for conspicuity reasons. The same detection errors have been documented during follow-up (8).

The variability in the selection of the diseased organ was greater for the NTL than for the TL, in 40% and 60% of patients, respectively. This is concordant with the literature (22). This greater variability demonstrates that the NTL category contains more ambiguous findings with respect to the TL lesions, which agrees with the literature.

**Figure 8:**
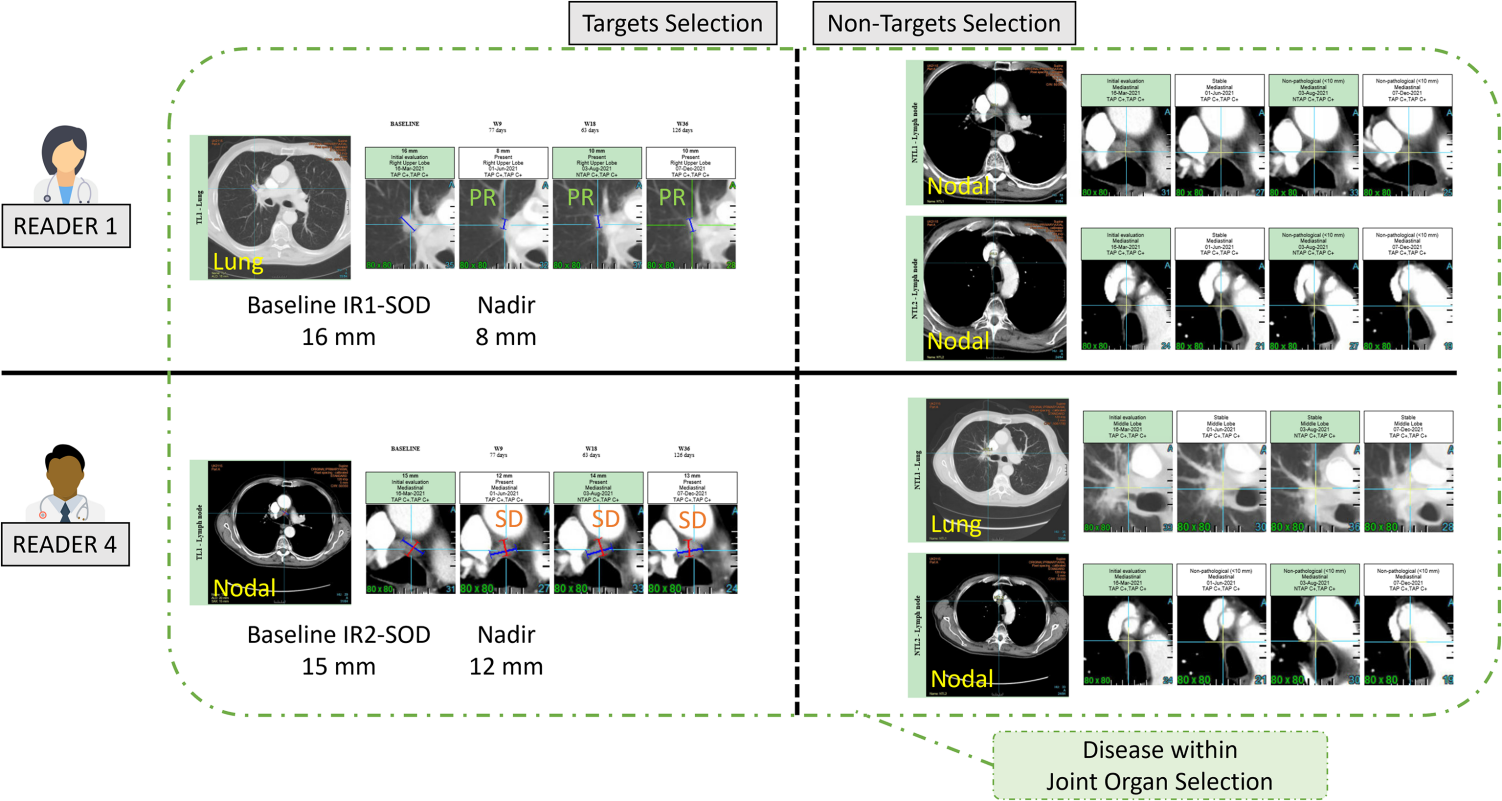
Example of inter-reader variability in classifying target and non-target lesions. In this example two readers (R1 and R4) selected the same lesions with different classification of target and non-target leading to discrepant responses. This discrepancy hinges on the measurement of the lymph node (15mm for Reader 4, and unstated for Reader 1) as well as the subjective opinion of Readers 1 and 4 about the measurability of the lung lesion.

Even when readers consider the same organ, classifying tumors as TL or NTL is of importance as some studies (15, 23) showed possible difference in malignancy, which consequently led to discordance in the evaluation of treatment response (22). A limitation in the RECIST 1.1 rules (20) may explain the origin of such discordances because, unlike the TL, the NTL category is designed to record the smallest measurable lesions and non-measurable lesions under 1** cm** (or short axis < 1.5** cm** for lymph node). Typically, a lung micro nodule may be considered as NTL by one reader while the paired reader may not consider the finding significant (see example in **Figure 8**) which could potentially lead to a discrepancy in evaluating the extent of disease. Indeed, during the follow-up, some of the differences between readers in capturing disease progression have been explained by possible dissociation of the response i.e., when tumors selected from different disease locations respond differently to treatment (24, 25). In trials evaluating the efficacy of immunotherapy, a dissociated response has been reported in 30% of patients for our indication (26). Discrepancies in baseline selection may increase the risk of discrepancies in double read evaluations if the patient experiences a dissociated response (27, 28).

### 5.4 Typology of radiologist readings

One of the challenges of BICR monitoring is to identify an **“**outlier**”** radiologist likely to increase the rate of discrepancies. The preferred key performance indicator (KPI) for this is based on follow-up radiological response timepoint (29). However, the performance of a reader who tends to under- or miss-diagnose the disease as early as baseline, could also be represented by specific performance indicators.

According to RECIST 1.1 criteria, the radiologist should collect up to five TLs with a maximum of two selected per organ. Our analysis showed that radiologists tended to select less than three TLs on average. This suggests that the representation of measurable disease of metastatic lung cancer is predominantly unifocal or bifocal. This is confirmed by the analysis in joint organ selection where the average number of TLs selected by both readers was also less than three for all trials (**Table 7**).

In addition, the violin plots in **Figure 5** shows that two radiologists (R1 and R6) tended to collect fewer TLs than their colleagues. In contrast, we observed that two readers (R3 and R5) tended to systematically select more TLs than other readers. As readers were involved in several studies, this demonstrates the existence of a specific reading pattern with a potential impact on double reading. The impact of a reader**’**s behavior on SOD is not straightforward. The distribution of tumor burden seemed globally similar for all the readers except two (R1 and R2) who had lower SOD measurements.

Comparing the selection profile of each reader helps to highlight the disease locations that are the most difficult to characterize, and which lead to greater inter-reader variability. We found there was a greater dispersion between readers on the recording of metastatic bone locations that are sometimes difficult to see, forgotten or ambiguous. Infrequent and therefore unexpected locations are also not surprisingly more dispersed and probably linked to their detection rate. The variability of reader**’**s evaluations in the context of lymph node disease remains important despite the greater frequency of this location. Two readers (R1 and R6) tended to record less disease in this location. This may again suggest the existence of a reading pattern specific to a radiologist.

Geijer et al. (30) documented the variability between two readers when they have differing experience, background, or interpretation of guidelines. As stated by Schmid et al. (5), “The greatest contributing factor of inter-reader variability originates from a radiologist’s own expertise”. In our study we observed a large inter-reader variability that would substantially contribute to the magnitude of the double read variability. The relationship between inter-reader and double read variabilities would require further investigation with the aim to optimize reader’s pairing.

### 5.5 Homogeneity of trials

We analyzed inter-trial homogeneity in a subset of patients for whom both readers selected the same disease locations for a more robust approach. Our analysis showed that disease presentation across trials differed significantly in terms of average size of TLs (ranging 28mm to 45mm) and tumor burden indicators. We reached same conclusions in focusing on the subgroup of lung and nodal TLs which are the most frequent targeted tumor locations. This variability is therefore a limitation of generalizing our KPI with the aim of benchmarking **“**comparable**”** metastatic non-small cell lung cancer (NSCLC) trials.

However, in concordant patients, the distribution of metastatic disease was comparable to the literature; readers primarily targeted the lung, lymph nodes, and more rarely, the liver and bone (14). The frequency of lymph node and bone disease was the most variable disease location across the trials, even when concordant evaluations were considered. Again, this shows that despite selecting patients according to relatively similar criteria, the presentation of the disease can differ greatly and may partly explain the differing rates of discordance in double readings found across the available literature for the same indication. The limitations in generalizing results across similar studies are related to the well-documented representativeness issues of the study population (31).

Although we endeavored to evaluate “comparable” trials, the patient population at each site may have had slightly varying characteristics (e.g., stage of the disease, treatment line). Our measurements showed inter-trial differences that can partially be explained by variable inclusion criteria. Liu et al. (32) did show that broadening restrictive inclusion criteria in advanced NSCLC trials had little impact on the trial hazard ratios, but little remains known about the impact of inclusion criteria and readers reliability (4).

### 5.6 Limitations

Firstly, our analysis of the reader~trial interaction was a partial analysis. We measured the variability of only two features (SOD and TLNum) as no recognized statistics were available to analyze the interaction within the measuring proportion (e.g. TopDisLocDisease). We were also limited by our data as all readers were not involved in all five trials and not all readers measurements were applicable to the different steps of our analysis.

Secondly, our raw data were blinded from tumor coordinates therefore, unlike Kuhl et al. (22), it was impossible to identify when the exact same finding was selected by both readers. The highest level of localization was at organ level.

Thirdly, as we were blinded from randomization, we were not able to refine our analysis by treatment or control. All trials included consisted of two sub-cohorts.

Fourthly, our two-way analysis considered only two features; the average number of TLs and SOD.

Finally, the analysis focused on a specific metastatic cancer indication thus limiting the generalization to another type of primary cancer. Indeed, our variability root cause analysis demonstrates that variability depends greatly on the metastasis locations known to be related to the primary cancer.

### 5.7 Perspectives

We proposed an innovative method that can be applied to clinical trials that use RECIST 1.1 to explore the initial disease presentation assessment and the variability of these assessments.

Bearing in mind the caution against generalization, our baseline variability analysis may help with detecting a deviation from an expected variability rate and lead to early investigation into the origin of the deviation. In the context of BICR, our baseline variability analysis can contribute to the quality control of reads. The double and single read-derived features should be further investigated for this purpose.

The initial investigation should consist of evaluating the correlation between the variabilities of feature values at baseline and at the corresponding therapeutic response evaluation timepoint. A significant correlation would allow to build a predictive model for the reliability of the evaluation using minimal data, therefore triggering early corrective actions or adaptations to trial sample size.

The second investigation should focus on features derived from single radiologist assessments. The existence of patterns attached to a set of radiologists would allow optimal pairing of radiologists for double reading.

The last application of our features applies to the core annotations (annotations performed on the same diseases by double reads); to confirm that clinical trials expected to be **“**similar**”** really are.

With the emerging use of synthetic arms (33), it has become very attractive to pool several **“**similar**”** control arms together to design a single synthetic larger one.

Ultimately, considering variabilities and discrepancies only as **“**an event to avoid**”** is probably not an optimal strategy. The baseline variability assessment is not purely noise. From a patient benefit perspective, a second opinion still means a higher chance of a correct diagnosis. The discrepancy event with the use of the proposed framework of analysis can help us detect both pseudo-lesions (34) at the baseline disease assessment and real metastasis missed by single reading.

As we can expect more information from combining readers**’** annotations into logical sets and applying the advanced algebra of our features, it may be possible to detect dissociated responses or to improve our understanding of the disease prognostic and drug mechanism of action.

## 6 Conclusion

Variability in baseline disease selection is known to be one of the major contributors to RECIST 1.1 inter-reader variability and is largely documented in previous papers.

Our analysis focused on the discrepancy between radiologists in disease selection. We provided an innovative method for quantifying discrepant tumor burden evaluations and for qualifying discrepant tumor distribution evaluations.

Considering our dataset extracted from five trials in metastatic NSCLC, we found that approximately 15% of patient tumor burden was measured in discrepant locations. The locations with the highest risk of discrepancies in disease identification were the lymph nodes and bone metastasis.

Our figures showed a greater uncertainty on the selection of the disease in the NTL category compared to the TL category.

The baseline lesion selection criteria in the RECIST guidelines leaves room for subjective assessments, potentially causing some of the observed differences in the chosen target or non-target lesions.

By analyzing the reader’s baseline assessments, we observed the existence of a reader’s specific pattern of assessment. This explains in part the observed inter-reader variability and could lead to possible pairing optimization to decrease discrepancies between readers.

In addition, we demonstrated that even though lung trials may be comparable in terms of the patient population, the indication, inclusion criteria and the primary tumor, that does not necessarily ensure their comparability in terms of disease presentation. Therefore, literature-based benchmarks for discrepancy KPIs should be used with caution.

## Data availability statement

The data analyzed in this study is subject to the following licenses/restrictions: Datasets are clinical trials sponsors properties. Requests to access these datasets should be directed to hubert.beaumont@mediantechnologies.com.

## Ethics statement

Ethical review and approval was not required for the study on human participants in accordance with the local legislation and institutional requirements. Written informed consent for participation was not required for this study in accordance with the national legislation and the institutional requirements.

## Author contributions

HB: Conceptualization, methodology, data curation, formal analysis, original draft, writing, review, and editing. AI: Conceptualization, methodology, formal analysis, project administration, original draft, review, and editing. All authors contributed to the article and approved the submitted version.
