# Supplementary material for: Breaking down the RECIST 1.1 double read variability in lung trials: What do baseline assessments tell us?
Source: Front Oncol. 2023 Mar 16;13:988784. doi: 10.3389/fonc.2023.988784 (PMC10060958; doi:10.3389/fonc.2023.988784)
Supplement: Supplementary file 1 [file DataSheet_1.docx]

Annexes

1. List of disease locations

| 1. Lung | 2. Liver | 3. Lymph node | 4. Pleura | 5. Chest wall |
| --- | --- | --- | --- | --- |
| 6. Bone | 7. Abdominal wall | 8. Adrenal gland | 9. Brain | 10. Spleen |
| 11. Bone marrow | 12. Esophagus | 13. Kidney | 14. Mediastinum | 15. Peritoneum |
| 16. Muscle | 17. Subcutis | 18. Pericardial. cavity | 19. Skin | 20. Pancreas |
| 21. Gastric | 22. Blood vessels | 23. Heart | 24. Neck | 25. Spinal cord |
| 26. Thyroid | 25. Diaphragm | 28. Pelvis | 29. Breast | 30. Trachea |

- An additional category, the 31st, labeled as “miscellaneous” was added
- Infrequent diseases are those from to 10 (spleen) to 31 (miscellaneous)

B. Detailed computing of the DisLocSOD feature

Considering:

SODi: Tumor burden as reported by reader i

C_SOD_i_: Part of the tumor burden that targets same organs selected by the other reader

S_SOD_i_: Part of the tumor burden that targets organs not selected by other reader

${SOD}_{i}={C\_SOD}_{i}+{S\_SOD}_{i}$ Equation 1

The specific SOD can be defined as:

$SPropSOD={100*{S\_SOD}_{i}/{SOD}_{i}}$ Equation 2

So that, when a reader targeted the same organs DisLocSOD=0 while when readers targeted completely different organs DisLocSOD= 1
